# Highly unstable complex C3-type distal femur fracture: can double plating via a modified Olerud extensile approach be a standby solution?

**DOI:** 10.1007/s10195-012-0204-0

**Published:** 2012-06-26

**Authors:** Ayman El-Sayed Khalil, Mostafa Ahmed Ayoub

**Affiliations:** 1 Department of Orthopaedic Surgery and Traumatology, Faculty of Medicine, Tanta University Hospital, University of Tanta, Al-Geish Street, Tanta, Egypt; 2 20 Taha Hussein Street, Al-Haram, Giza, Egypt

**Keywords:** Highly unstable C3-type distal femur fracture, Extensile approach, Distal femur double plating, Multiplanar femoral condyle fractures

## Abstract

**Background:**

Multiplanar complex C3-type unstable distal femoral fractures present many challenges in terms of approach and fixation. This prospective study investigates a possible solution to these problems through double plating with autogenous bone grafting via a modified Olerud extensile approach.

**Materials and methods:**

Twelve patients with closed C3-type injuries were included; eight of them were male, and their mean age was 33.5 years (range 22–44 years). Mechanism of injury was road traffic accident (RTA) in nine patients and fall from height in the other three cases. Eight cases were operated during the first week and four cases during the second week after injury. Mean follow-up was 13.7 months (range 11–18 months).

**Results:**

Mean radiological healing time was 18.3 weeks (range 12-28 weeks), and all cases had good radiological healing without recorded nonunion or malunion. Clinically, two cases (16.7 %) had excellent results, five cases (41.7 %) had good results, three cases (25 %) had fair results, and two cases (16.7 %) had poor results. No cases developed skin necrosis, deep infection, bone collapse, or implant failure. However, two cases (16.7 %) had limited knee flexion to 90° and required subsequent quadricepsplasty.

**Conclusions:**

Use of this modified highly invasive approach facilitated anatomical reconstruction of C3-type complex distal femoral fractures with lower expected complication rate and acceptable clinical outcome, especially offering good reconstruction of the suprapatellar pouch area. It can be considered as a standby solution for managing these difficult injuries.

## Introduction

Treatment of comminuted distal femur fractures is problematic [1]. They are often difficult to treat because they usually result from high-velocity trauma, producing comminution, bone loss, and an unstable fracture pattern [2]. Müller et al. [3] classified these fractures according to their location and pattern. Their classification broadly divides distal femoral fractures into type A (extraarticular with three subtypes), type B (condylar or partial articular with three subtypes), and type C (bicondylar or complete articular with three subtypes). Among these, C3-type fracture entails significant articular comminution with fractures in all planes and remains the most difficult surgical challenge.

Three main problems are commonly observed in these fractures. First, adequate exposure of articular surface, particularly of medial femoral condyle and coronal plane fractures, is exhausting. Second, the standard implants used for other types of distal femoral fracture such as the condylar blade plate and supracondylar nails are not helpful for articular surface reduction and fixation. Third, in setting of medial comminution and short distal segment, there is high incidence of loss of fixation and varus collapse [2, 4]. Accordingly, this prospective study evaluates the results of double plating of highly unstable C3-type multiplanar distal femur fracture through a modified Olerud extensile approach.

## Materials and methods

Between January 2007 and October 2010 we treated 12 polytraumatized adult patients with closed comminuted distal femur fractures using a lateral distal femur locked plate and a medial contoured plate through a modified Olerud extensile approach. All patients gave informed consent prior to being included in the study, and the study was authorized by the local ethical committee and performed in accordance with the ethical standards of the 1964 Declaration of Helsinki as revised in 2000. All patients had C3-type fracture according to the classification of Muller et al. [3]. The exclusion criteria included all other types of distal femur fracture, as they can be addressed easily with ordinary or mini-invasive lateral or lateral parapatellar approaches without the need for osteotomy of the tibial tubercle. In all patients, the fracture lines within the condyles ran in sagittal, coronal, and oblique planes in addition to metaphyseal comminution of variable degree. All cases had associated injuries as summarized in Table 1; the mean Hannover polytrauma score (PTS) was 29.8 points (range 22–36 points). Eight cases were males, and five of them were heavy smokers. Five cases were overweight and two cases were obese, as shown in Table 2. Mean age was 33.5 years (range 22–44 years). Road traffic accidents (RTA) were responsible for the injuries in nine patients, and the other three cases had history of fall from height. The distal neurovascular state of the affected extremity was intact in all patients, as evidenced clinically in nine of them and through vascular surgeon consultation in the other three questionable cases. Timing of surgery varied according to the associated injuries and the local soft tissue condition of the distal femur. However, eight cases were operated upon during the first week and four cases during the second week after injury. Plain anteroposterior (AP) and lateral X-rays views were taken, and if needed oblique views, to classify the injury, map the fracture lines, and help with preoperative surgical planning in each case. Computed tomography (CT) scans and their three-dimensional (3-D) reconstruction were mandatory after the fourth case due to preoperative missing of associated Hoffa fractures in three of them, for more meticulous surgical planning, and for medicolegal purposes. However, Hoffa fracture was encountered in seven cases, with involvement of the lateral condyle in four cases and the medial one in three cases. Chemical prophylaxis for deep vein thrombosis with daily subcutaneous dosage of 40 mg enoxaparin, a low-molecular-weight heparin (LMWH), was applied in all cases; it was initiated for an average of 5.3 weeks (range 3–7 weeks), and continued thereafter with daily dosage of 20 mg until full weight-bearing was achieved.

**Table 1:** Associated injuries

Associated injuries	Number	Percentage
Minor head injuries	5	41.7
Chest injuries	10	83.3
Abdominal injuries	7	58.3
Upper limb injuries	4	33.4
Ipsilateral lower limb injuries	2	16.7
Contralateral lower limb injuries	4	33.4
Stable pelvic fractures	5	41.7
Stable spinal fractures	3	25

**Table 2:** Patient demographic data

Patient no.	Sex	Age (years)	Body weight	Smoking history	Mechanism of injury	PTS* points	Fracture type	Side involved	Knee derangement	Timing of fixation	Approach-related complications	Fracture-related complications	Range of motions (degrees)	Functional results**
1	Male	28	Obese	+ve	Pedestrian	32	C3	Left	Present	Second week	Delayed healing	Delayed union	0–110	Fair
2	Male	26	Over	+ve	Fall from height	24	C3	Right	Absent	First week	Absent	Delayed union	0–116	Good
3	Female	31	Normal	−ve	Pedestrian	33	C3	Right	Absent	Second week	Superficial infection	Absent	0–124	Good
4	Male	37	Over	−ve	Motorcycle	32	C3	Left	Present	First week	Delayed osteotomy healing	Absent	0–95	Fair
5	Female	34	Normal	−ve	Car occupant	36	C3	Right	Absent	Second week	Delayed healing	Absent	0–113	Good
6	Male	43	Over	+ve	Fall from height	27	C3	Left	Present	First week	Absent	Delayed union	0–110	Fair
7	Male	39	Obese	−ve	Motorcycle	26	C3	Left	Present	First week	Delayed osteotomy healing	Absent	0–90	Poor
8	Female	41	Over	−ve	Pedestrian	34	C3	Right	Absent	First week	Absent	Absent	0–120	Good
9	Female	44	Normal	−ve	Fall from height	22	C3	Right	Absent	First week	Absent	Absent	0–130	Excellent
10	Male	25	Over	+ve	Motorcycle	35	C3	Left	Present	Second week	Superficial infection	Delayed union	0–90	Poor
11	Male	32	Under	+ve	Car occupant	29	C3	Right	Absent	First week	Absent	Absent	0–121	Good
12	Male	32	Under	−ve	Motorcycle	28	C3	Right	Absent	First week	Absent	Absent	0–130	Excellent

* Hannover polytrauma score, ** Clinical scoring of Sanders et al. [2]

**Fig. 1:**
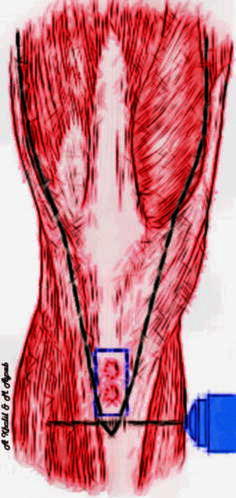
Diagram showing the proposed V-shaped skin incision, tibial tuberosity osteotomy with two screws in predrilled holes, and transverse tunnel for tension band wiring

**Fig. 2:**
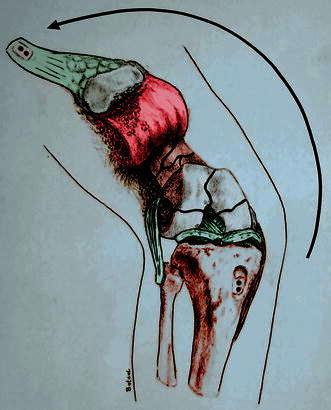
Diagram showing the comminuted distal end of femur after finishing the approach with upward reflexion of the whole extensor mechanism including the osteotomized tibial tuberosity

**Fig. 3:**
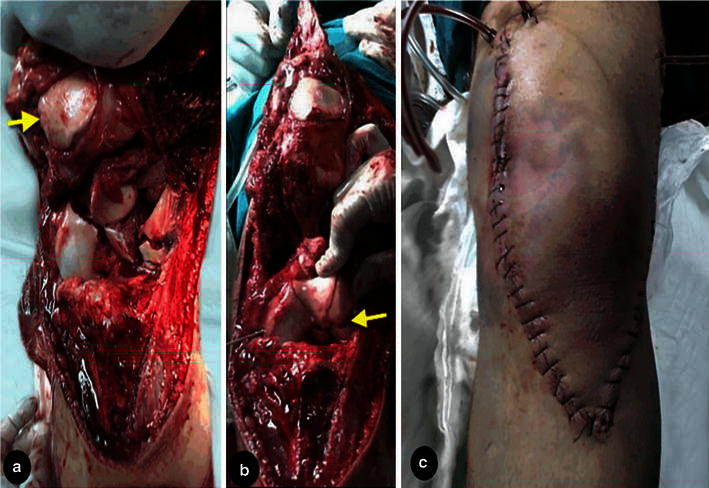
**a** Case 4: intraoperative photograph showing the left-sided multiplanar comminuted C3-type fracture with *arrow* indicating the reflected patella with intact suprapatellar pouch area after finishing the modified Olerud approach. **b** Same case: intraoperative photograph after assembly of the distal end of femur and temporary K-wire fixation; *arrow* indicates Hoffa fracture of the lateral femoral condyle. **c** Same case: photograph showing meticulous wound closure with applied medial and lateral suction drains

At time of the planned surgery, prophylactic parenteral antibiotic was given. The operation was carried out with the patient lying supine, with a pillow under the knee to allow 90° knee flexion. A thigh tourniquet was used if the fracture did not extend too far proximally, being released before wound closure to control bleeding. The ipsilateral iliac bone was draped for autografting. The extensile approach as prescribed by Olerud was applied in all cases [5]. We modified the approach at certain points (Figs. 1, 2). First, the Y-shaped skin incision was replaced with our V-shaped incision with its apex just 1 cm below the tibial tuberosity. Second, two predrilled holes were created in the tibial tuberosity to allow fixation with two washered 6.5-mm cancellous screws at the end of the procedure. Third, the tibial tuberosity was marked with electrocautery, and its osteotomy was initiated with electric saw and completed with sharp osteotome. Fourth, the suprapatellar pouch was preserved as much as possible in the undersurface of the reflected extensor mechanism. Fifth, tension band wiring, over the head of the proximal screw and through a transverse tunnel distal to the osteotomy site, was added to augment fixation. After complete approach, debridement, and fracture fragment assembly, preliminary Kirschner wires (K-wires) were applied smoothly from all aspects around the exposed distal end of the femur (Fig. 3a, b). Definitive fixation began with countersunk cannulated cancellous screws, including those for Hoffa fractures, then lateral locked distal femur plate application, and finally application of the contoured medial plate (reconstruction plate in eight cases, semitubular plate in four cases). Finally, autogenous iliac bone grafting was used to fill all bony defects with good impaction, especially in the anterior and medial aspects, followed by meticulous closure of the wound (Fig. 3c).

Postoperatively, the limb was placed in a hinged knee immobilizer. Unfortunately, a continuous passive machine (CPM) was not available, but the hinges were unlocked to allow assisted gradual full range of motion under physiotherapy control. The range of motion started at 30° and was then advanced on a daily basis. Underarm-crutch-assisted partial weight-bearing was progressive, with full weight-bearing being postponed until there was radiographic evidence of bony union (minimum of 12 weeks postoperatively). Mean follow-up was 13.7 months (range 11–18 months).

At the end of the follow-up period, functional results were evaluated according to the method of Sanders et al. [2], which depends on range of motions (0–9 points), pain (0–10 points), deformity (0–6 points), walking ability (0–9 points), and return to work (0–6 points) as parameters for clinical scoring. Accordingly, patients with 36–40 points, 26–35 points, 16–25 points, or 0–15 points had excellent, good, fair, or poor results, respectively.

Statistical analysis was done using SPSS version 11.0.1 for Windows (SPSS Inc., Chicago, IL). One-way analysis of variance (ANOVA) and its nonparametric equivalent, the Kruskal–Wallis test, were used for variables which were small and not normally distributed. *P* value ≤ 0.05 was considered statistically significant.

## Results

All our cases had good radiological healing without recorded nonunion or malunion. Reductions were near anatomic (<2 mm step-off, <5° varus, <1 cm shortening) in all patients. Mean radiological healing time was 18.3 weeks (range 12–28 weeks), as four cases (33.3 %) had union delayed for more than 24 weeks.

**Fig. 4:**
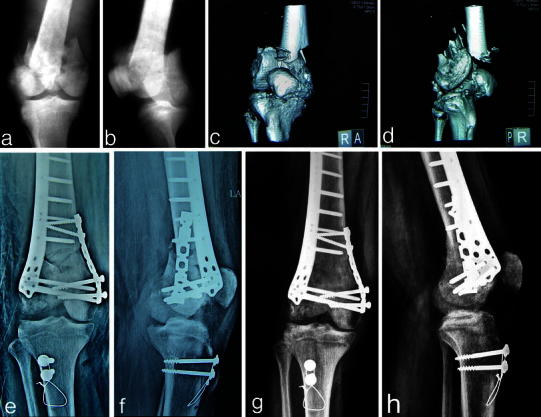
**a**, **b** Case number 12: AP and lateral plain X-ray views showing typical C3-type distal right femur fracture. **c**, **d** 3-D reconstruction, AP and lateral CT scans. **e**, **f** Immediate postoperative views showing the lateral locked condylar plate and the medial contoured reconstruction plate with its distal 6.5-mm cancellous screws with prominent heads due to mandatory obliquity of the screws inside the bone. **g**, **h** The same views after 9 months, showing complete radiological healing of fracture in good anatomical position with incorporation of the metaphyseal autogenous bone grafts and excellent healing of the tibial tuberosity osteotomy without loosening of the implants. The medial prominent screw heads did not bother the patient or decelerate the early aggressive rehabilitation program. The patient returned fully to his previous work with painless 130° knee flexion and no extensor lag

**Fig. 5:**
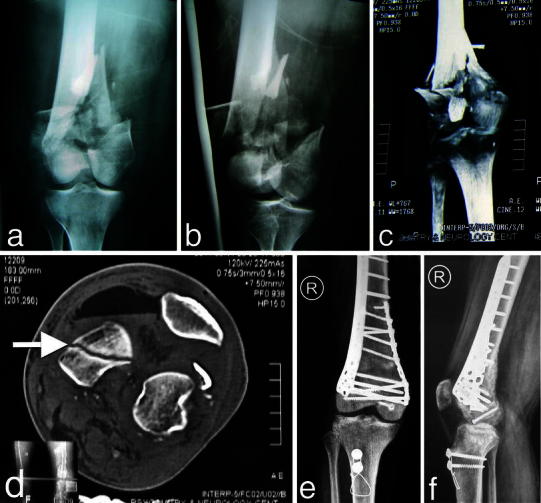
**a**, **b** Case number 11: AP and lateral plain X-ray views showing C3-type distal right femur fracture. **c** The related 3-D reconstruction anteroposterior CT scan. **d** The axial CT scan: distal cut showing medial Hoffa fracture (*white arrow*). The fracture was fixed by a lateral locked condylar plate, a medial contoured reconstruction plate, and a countersunk screw for the medial Hoffa fracture. **e**, **f** AP and lateral views 1 year after surgery, showing good union. The patient regained about 120° of knee flexion without any extensor lag

Clinically, two cases (16.7 %) had excellent results (Fig. 4), five cases (41.7 %) had good results (Fig. 5), three cases (25 %) had fair results, and two cases (16.7 %) had poor results. The unsatisfactory (fair and poor) results were due to restricted knee motions in all five cases, in addition to pain of variable degree and walking disability in three of them, and change of the original work in four of them.

Using our modified method, no cases presented skin necrosis or deep infection. However, controlled superficial infection was recorded in two cases (16.7 %), delayed wound healing was present in another two cases (16.7 %), and delayed tibial tuberosity osteotomy healing for more than 12 weeks was encountered in two cases (16.7 %), as presented in Table 2. They had insignificant effect on the final clinical outcome (*P* = 0.600, Kruskal–Wallis test).

Mean duration of tibial tuberosity osteotomy healing was 10.5 weeks (range 8–14 weeks), and no case had implant loosening, failure, or nonunion. All cases had mild objective infrapatellar paraesthesia which was not agonizing subjectively for any of them at end of follow-up.

The variables studied in our series had insignificant effect on the clinical outcome due to the small number of cases. However, the results were (insignificantly) better among: females, underweight and normal-weight patients, nonsmokers, those with fall-from-height injuries, cases operated during the first week, and patients without typical Hoffa fractures or associated insult of cruciate ligament femoral attachment, as presented in Table 3.

**Table 3:** Variables affecting functional outcome

Variables	Functional results					Statistical analysis	
	Excellent cases	Good cases	Fair cases	Poor cases	Total	*P* value	Test related
Gender							
Male	1	2	3	2	8	0.686*	Kruskal–Wallis test
Female	1	3	0	0	4		
Age groups (years)							
21–30	1	1	1	1	4	0.334*	Kruskal–Wallis test
31–40	0	3	1	1	5		
41–50	1	1	1	0	3		
Weight							
Underweight	1	1	0	0	2	0.517*	One Way Analysis of Variance
Normal	1	2	0	0	3		
Overweight	0	2	2	1	5		
Obese	0	0	1	1	2		
Smoking							
Smokers	0	2	2	1	5	0.476*	Kruskal–Wallis test
Non-smokers	2	3	1	1	7		
Mechanism of injury							
RTA**	1	4	2	2	9	0.838*	Kruskal–Wallis test
Fall from height	1	1	1	0	3		
Timing of internal fixation							
First week	2	3	2	1	8	0.476*	Kruskal–Wallis test
Second week	0	2	1	1	4		
Knee derangement							
Absent	2	5	0	0	7	0.952*	Kruskal–Wallis test
Present	0	0	3	2	5		
Hoffa fractures							
Present	0	2	3	2	7	0.705*	Kruskal–Wallis test
Absent	2	3	0	0	5		

* Statistically insignificant, ** Road traffic accidents

In addition to the above-mentioned residual knee stiffness, approach-related complications, and delayed fracture union, three cases (25 %) had mild pain at the iliac grafting donor site and two cases (16.7 %) had manipulation under general anesthesia after 3 weeks from surgery due to manifest delay in rehabilitation response and fear of development of arthrofibrosis. The five cases with cruciate ligament insult had objective anteroposterior laxity of only ±1 without subjective instability or giving way. The two cases (16.7 %) with severe restriction of knee flexion had initially severe injury to the suprapatellar pouch area, and they sought quadricepsplasty surgery after 14 and 16 months, respectively.

## Discussion

C3-type distal femur fracture presents a challenging problem to orthopedic surgeons. Distal femoral nails cannot address these injuries due to the low fracture lines with articular comminutions, and the need for free multidirectional lag screws for prior assembly of the fragments that would interfere with insertion of these nails. Therefore, Kim et al. [6], with less severe injuries, stated that poor results, nail failure, and complications were related to the low fracture lines with comminution. Also, Pröbstel and Börner [7] declared that retrograde nailing achieves early consolidation without primary bone grafting but is complicated by a greater amount of instability and malalignment in the presence of comminutions. Ilizarov external fixators with minimal internal fixation have many disadvantages in managing C-type injuries including imperfect reduction, septic arthritis, osteomyelitis, pin tract infection, loss of reduction, delayed union or nonunion requiring bone grafting, and limited knee motion, requiring manipulation under anesthesia or quadricepsplasty, due to spanning of the knee in ordinary apparatus and tethering of the quadriceps muscles in the low-profile apparatus [8–12].

Olerud [5] described his extensile approach with its Y-shaped skin incision for these difficult injuries, and in spite of skin healing problems, use of ordinary lateral angled blade plate, absence of medial plating, infection in four cases, and single screw fixation of the tibial tuberosity osteotomy, he reported satisfactory overall clinical outcome in his study. To manage these injuries, Sanders et al. [2] also added a medial buttressing plate for their nine cases, and in spite of their rigid fixation and early rehabilitation, three patients had <90° flexion, and in six the arc of flexion was limited to between 90° and 100°. Additionally, four patients had extensor lag of 5°.

In this series, we tried to overcome most of the problems related to these severe injuries by internal fixation through modification of the extensile Olerud approach, anatomical reduction of the multiplanar fractures including medial and lateral Hoffa fractures, use of a locked distal femur plate, augmentation of the bony defects by medial buttress plating and bone grafting, assembly of the fragments related to the femoral attachments of the cruciate ligaments, preservation of suprapatellar pouch integrity, and early aggressive rehabilitation. The V-shaped skin incision in our cases precluded the wound-edge necrosis and dehiscence encountered by Olerud with the Y-shaped incision. The rigid fixation of the tibial tuberosity osteotomy by two screws and tension band wiring encouraged the early and progressive physiotherapy program.

Similar to literature findings and in spite of the extensile approach, late bone collapse secondary to blood supply impairment did not occur in this series [5, 13]. This may be due to the extensive primary bone grafting and the fact that the blood supply of the bone in this area is mainly posterior through the major soft tissue attachments.

Although three Hoffa fractures were missed during preoperative plain radiological evaluation alone, anatomical reduction and fixation was achieved smoothly in all of them using the described approach. However, Hoffa fractures were accompanied with less satisfactory results; this may be due to the large articular defect following the countersinking technique, which could not be covered completely by regenerated fibrocartilage. Borse et al. [14] advised use of headless compression screws to reduce this fixation problem. Also, we agree with Baker et al. [15] that computed tomography is extremely helpful for characterization of complex intraarticular fractures of the distal femur and in diagnosis of missed Hoffa fractures.

Associated cruciate ligament femoral attachment injuries did not significantly affect the clinical results in the study, which may be related to the small number of cases, and the sound healing after reduction and fragment fixation with residual objective anteroposterior instability of only ±1.

The locked distal femur plate played an important role in fixation in this study, especially in the presence of severe medial comminution. However, it has the major disadvantage of uniaxial screw direction, which was overcome by prior application of separate lag screws. Biomechanically, Koval et al. [16] found that the locked buttress plate provided significantly greater fixation stability than the standard plate or blade plate, both before and after cycling in axial loading. The application of the medial buttress plate in this study increased the fixation construct rigidity, facilitated graft impaction, and encouraged early rehabilitation without loss of reduction. Also, Sanders et al. [2] reported the efficacy of a medial plate and bone graft in maintenance of reduction during loading in early active motion without loss of reduction or loosening of the implant. Moreover, Jazrawi et al. [1] found that the locked, double-plate construct provided significantly greater fixation stability than the standard double-plate construct.

No case with nonunion was encountered among our cases, which might be due to the absence of open injury cases, the primary autogenous bone grafting, and the biological fixation using the locking plate with preservation of periosteal blood supply as documented by Gwathmey et al., Perren, and Lujan et al. [4, 17, 18].

In spite of the extensile approach and the expected high rate of deep infection in this situation, no case developed this disastrous complication in this study. This could be due to the absence of open injuries, use of prophylactic antibiotic, and the meticulous soft tissue dissection. Similarly, Mize et al. [13], using their extensile approach, applied the same principle in eight of their cases with satisfactory clinical results and no recorded infection or skin problems among them.

Stiffness of the knee with maximum 90° flexion involved two cases (16.7 %) in this series. They had initially severe suprapatellar pouch injury with massive anteromedial comminution. This incidence of stiffness is lower than found in the literature (25–33.3 %) for extensile approaches [2, 5, 13]. This could be due to the preservation of the whole suprapatellar sac in this modified approach, anatomical restoration of the distal end of femur with grafting of voids, rigid internal fixation, and early aggressive rehabilitation. Likewise, Ziran et al. [19] felt that extensive damage to suprapatellar tissues and lack of immediate early motion contributed to fibrosis and stiffness.

Reviewing the literature, there are no reports strictly on the approach and management of C3-type distal femur fractures. However, all recent reports concern less-invasive lateral or lateral parapatellar approaches with locked plate application to all types of distal femur fractures, most of them being of type A, B, C1, and C2, plus a few C3-type injuries [20–22]. In spite of this, many authors were forced to do tibial tubercle osteotomy to facilitate reduction and fixation of these complex injuries [21, 23, 24]. Moreover, Dhillon et al. [25] were obligated to adopt a separate medial approach for managing medial Hoffa fractures. Therefore, this study approach can be suggested in difficult C3-type cases when other approaches necessitate tibial tubercle osteotomy to anatomically reconstruct comminuted distal condylar femur fractures.

Although use of a distal femur lateral locking plate with or without application of lag screws has been the gold standard during the last decade, most recent works reported high rates of defective callus formation, delayed union, nonunion, malrotation, and implant failure, especially in comminuted cases, despite use of a single ordinary or mini-invasive lateral or anterolateral approach [26–30]. This could be due to a mechanical problem related to application of this plate, particularly in the absence of fragment compression and primary autogenous bone grafting. In this study, the lateral locked plate was applied in all cases, with 100 % union rate, which could be attributed to the easily applied isolated multidirectional compression lag screws, the medial support plating, and the primary autogenous bone grafting, although it added a second site of potential morbidity in 25 % of patients.

Regardless of the small number of cases as a shortcoming of this study, we found that the modified Olerud approach is a useful alternative approach for complex C3-type distal femur fracture when single or double separate medial and lateral approaches without tibial tubercle osteotomy are not sufficient for anatomical reconstruction of the comminuted distal femoral condyles. It had the advantages of: complete and anatomical reconstruction of these severe injuries, facilitation of preliminary K-wire fixation from all directions around the distal end of femur, preservation of the whole suprapatellar pouch in the undersurface of the reflected extensor mechanism, comfortable application of the medial plate, ideal fixation of medial and lateral Hoffa fractures, complete grafting of bony defects at all locations with good impaction, addressing associated internal knee derangement whenever possible, lower incidence of suprapatellar area adhesions, and uncomplicated wound healing. We believe that it will also be highly valuable in revision surgery after implant failure and nonunion of C3-type injuries, and in addressing comminuted distal femur fracture combined with ipsilateral displaced tibial plateau fracture, although these were not encountered in this series. We believe that future application of this modified Olerud approach in a larger number of C3-type distal femur fracture cases will reveal many other advantages and disadvantages not apparent in this study.
